# Acute Severe Calculous Cholecystitis with Multiorgan Failure Complicated by Scrub Typhus

**DOI:** 10.1155/2019/7505108

**Published:** 2019-06-24

**Authors:** Suman Acharya, Jayant Kumar Yadav, Nischal Khanal, Raju Bhandari, Bikal Ghimire

**Affiliations:** ^1^Tribhuwan University Teaching Hospital, Kathmandu, Nepal; ^2^TUTH Boys Hostel, Maharajgunj, Kathmandu, Nepal

## Abstract

Scrub typhus is a febrile illness and can present with manifestations ranging from subclinical symptoms to multiorgan failure and death. Scrub typhus is a rare etiology of acute cholecystitis. A patient presenting with the features of acute cholecystitis who does not respond to standard treatment should be screened for scrub typhus in a typhus endemic region. We report a case of a 70-year-old female with acute severe calculous cholecystitis with multiorgan failure complicated by scrub typhus. She improved remarkably after starting doxycycline for scrub typhus. Scrub typhus should be considered as a trigger in a patient presenting with cholecystitis in a typhus endemic region.

## 1. Introduction

Scrub typhus is a mite-borne acute febrile illness caused by Orientia tsutsugamushi. A patient usually presents with acute or insidious onset of fever with chills, malaise, headache, and myalgia. Rash, eschar, lymphadenopathy along with other signs and symptoms may be present. A patient with severe infection may present with complications like acute kidney injury (AKI), acute respiratory distress syndrome (ARDS), meningoencephalitis, myocarditis, and death [1, 2]. Scrub typhus has been reported regularly from Nepal. In 2016, 831 cases were reported from 47 out of 75 districts of Nepal mostly from the southern part [3]. However, it is often underrecognized among clinicians in Nepal, and deaths have been reported due to delayed diagnosis. Scrub outbreaks have been frequently reported in the aftermath of the earthquake in Nepal [4–7]. Cholecystitis as a complication of scrub typhus is very uncommon and sparsely reported in the literature.

We report a case of a 70-year-old woman suffering from scrub typhus who presented to us with the features of acute cholecystitis and multiorgan failure.

## 2. Case Presentation

A 70-year-old woman presented to the emergency room of a university hospital with severe right upper quadrant abdominal pain without any radiation. It was associated with fever, nausea, and several episodes of vomiting for the past 4 days. There was no association of pain with the intake of food. She had not passed stool for the last 3 days and also complained of abdominal fullness for the past 2 days.

On examination, the patient appeared drowsy, ill-looking with a pulse of 78 bpm, blood pressure of 140/100 mmHg, respiratory rate of 18 min^−1^, a temperature of 100°F, and oxygen saturation of 74% under room air. The patient was anicteric. Voluntary guarding was present on abdominal examination and tenderness was present on the right upper quadrant. Murphy's sign was positive. Normal bowel sounds were audible. Systemic examinations were within normal limits. She had no other comorbidities.

Laboratory investigation revealed neutrophilic leukocytosis with total leukocytic count 12600 mm^−3^ and 82% neutrophil. Creatinine level was raised (260 *μ*mol/l). Liver function tests, serum amylase and lipase, hemoglobin, platelets, and coagulation profile were within normal limits. Arterial blood gas showed metabolic acidosis (pH 7.21, HCO_3_ 17.4, pCO_2_ 43.5, BE -9.1, and Lac 0.6). Ultrasonography revealed multiple cholelithiasis with distended gallbladder, and a pericholecystic collection was noted.

She was admitted with the diagnosis of severe acute cholecystitis and started on ceftriaxone and metronidazole along with other supportive medications.

On the 2nd day of admission, her urine output dropped and she became oliguric. Her blood pressure and pulse escalated up to 170/100 mmHg and 100 bpm, respectively. She was started on amlodipine 10 mg and intravenous labetalol. Investigations revealed deteriorating renal function with creatinine 416 *μ*mol/l along with worsening metabolic acidosis (pH 7.18). She was shifted to the intensive care unit and was started on imipenem/cilastatin.

**Figure 1:**
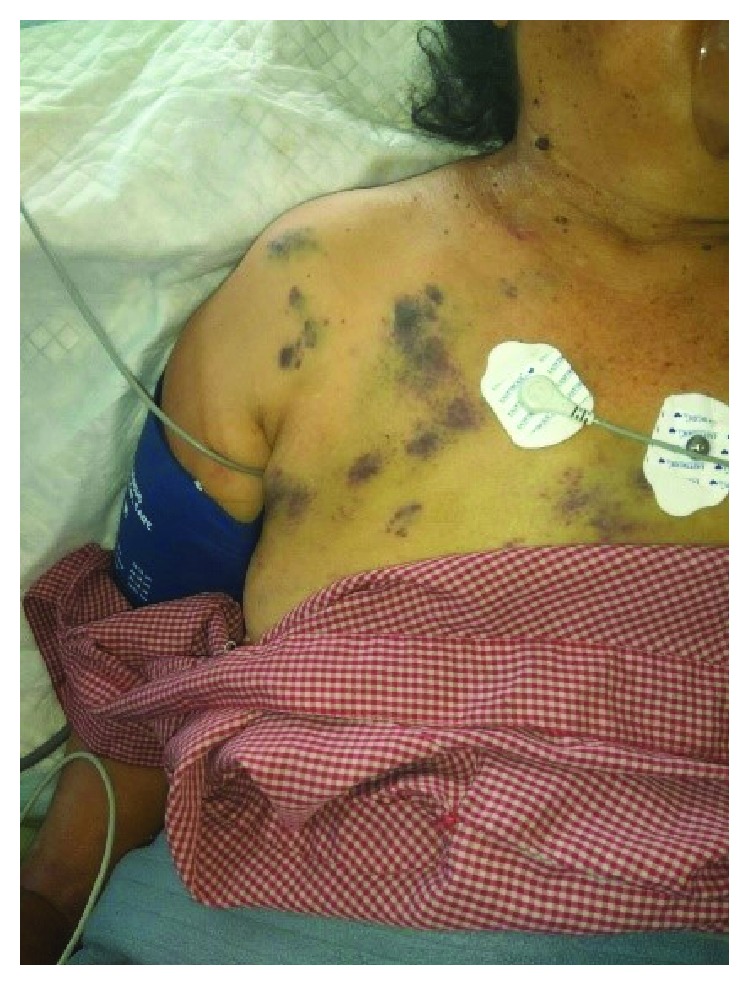
Purpuric rashes developed on the anterior chest and anterior abdomen after the patient was admitted.

On the 3rd day of admission, she was intubated for severe respiratory distress with worsening metabolic acidosis. Her blood pressure kept on rising (SBP up to 200 mmHg, DBP up to 115 mmHg) and was started on intravenous glyceryl trinitrate (GTN) infusion. Her hematological parameters were similar except for the declining level of platelets (93000 mm^−3^) on the 3rd day of admission. Urgent cholecystostomy was performed, and 100 ml of nonpurulent collection was drained and sent for culture. We identified purpuric rashes as well as few maculopapular rashes on the anterior chest and abdomen after she was admitted (Figure 1).

On the 5th day, development of new onset rash and an epidemiological clue led us to the workup for scrub typhus. However, eschar was not present. A rapid IgM antibody detection test performed by an InBios Scrub Typhus Detect™ test kit was positive for scrub typhus. Meanwhile, culture report showed no growth. The patient was started on doxycycline 100 mg twice daily on the same day. She underwent one cycle of hemodialysis for raised creatinine level and worsening metabolic acidosis.

**Figure 2:**
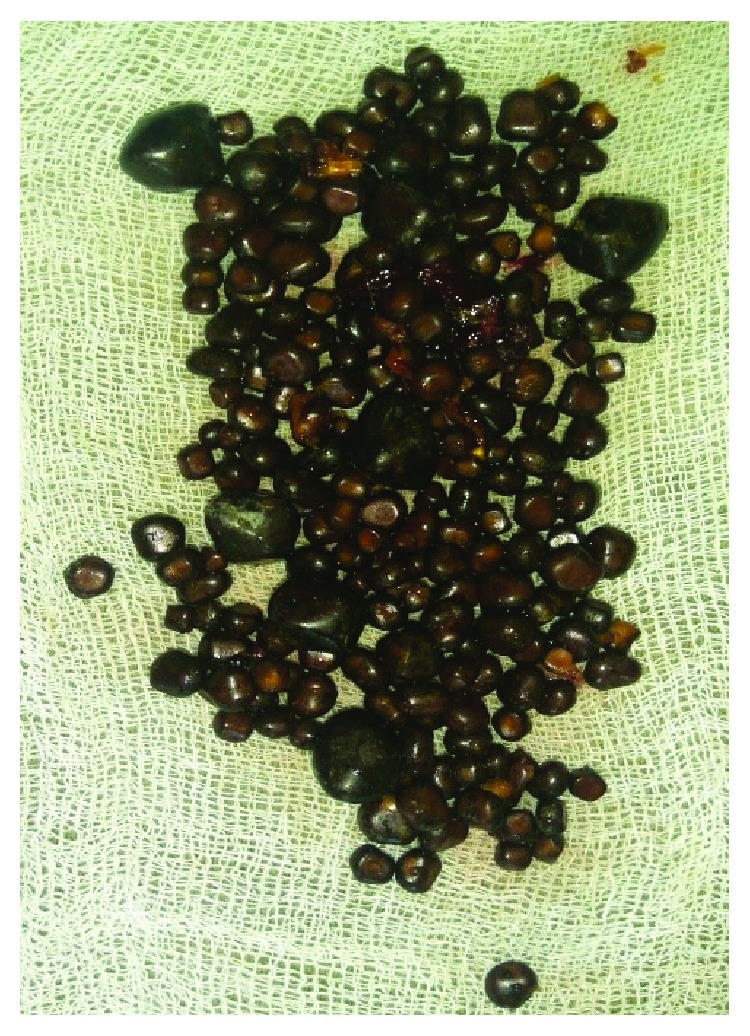
Laparoscopic cholecystectomy revealed multiple small calculi inside the gallbladder.

On the 6th day, the patient showed signs of improvement. Her hematologic parameters started improving. She was extubated and was shifted to intermediate critical care on the 6th day. Two days later, she was shifted to the general ward and was discharged on the 10th day of her admission. She underwent laparoscopic cholecystectomy 8 weeks later which revealed multiple calculi on her gallbladder (Figure 2). Her follow-up was uneventful.

## 3. Discussion

Acute cholecystitis usually occurs as a complication of gallstone disease especially in those who have a history of symptomatic gallstones. However, it may occur in those without gallstones. Cystic duct obstruction, increase in the levels of inflammatory mediators, and infection of the bile have been implicated in the pathogenesis of acute cholecystitis. Antibiotics, pain management, and gallbladder drainage are the recommended therapy for severe cholecystitis [8]. Left untreated, the gallbladder may become gangrenous and may cause perforation peritonitis.

The diagnosis of scrub typhus based on clinical symptoms is difficult; hence, the laboratory tests are needed to confirm the diagnosis. Immunochromatographic test kits are rapid and reliable tools for point-of-care serological testing in resource-limited settings. A recent meta-analysis has reported high sensitivity (>80%) and specificity (>90%) of the InBios Scrub Typhus Detect™ test kit [9, 10].

Cholecystitis as a complication of scrub typhus is a rare entity. In this case, acute cholecystitis could not be attributed to cholelithiasis alone as the patient continued to deteriorate despite treatment with antibiotics and gallbladder drainage, which is standard in the management of acute cholecystitis. However, the addition of doxycycline 100 mg twice a day to the regimen resulted in rapid improvement in the patient which is the standard management of typhus. Although rare, both calculous and acalculous cholecystitis as a complication of scrub typhus are reported in the literature [2, 11–14]. In a hospital-based study in India, 20 out of 330 patients developed acute cholecystitis as a complication of scrub typhus [15]. In our case, a gallbladder stone was present and cholecystitis was perhaps triggered by typhus infection.

The pathogenesis of cholecystitis in scrub typhus has been attributed to the development of vasculitis and perivasculitis [11, 16]. Further, vasculitis can also produce skin rashes which were present in the above case. Maculopapular rashes have been reported to appear at the end of the first week starting from the trunk and later spreading to limbs [17]. Presence of eschar, a localized necrotic lesion which appears at the site of the chigger bite, offers a clue to the diagnosis of scrub typhus. However, its presence is highly variable and may be present in 3-97% of cases and may be difficult to recognize in dark-skinned South Asians [1, 7]. Systemic hypertension in the above case could be attributed to disseminated vasculitis which got better along with management of scrub typhus.

Poor response to standard treatment for acute cholecystitis, confirmation of scrub typhus with a rapid IgM test, and quick recovery following introduction with doxycycline suggested that scrub typhus may have complicated the patient condition.

Lee et al. reported a longer hospital stay in patients who suffered from acute cholecystitis complicated by scrub typhus compared with controls who suffered from acute cholecystitis alone [11]. Timely diagnosis and commencement of proper medication can speed up the recovery in cholecystitis complicated by scrub typhus.

One of the key learning points was that scrub typhus may affect multiple organs and the gallbladder is not exempt. In an endemic region, scrub typhus should always be considered as a differential in a patient with severe cholecystitis who does not improve despite standard treatment.

## 4. Conclusion

The clinical manifestations of scrub typhus can vary from mild infection with fever, headache, and malaise to severe infection resulting in multiorgan failure and death. In an endemic region, a high index of suspicion is required for diagnosis and treatment in any patient who presents with fever and rash. We should bear in mind that acute cholecystitis can be a presenting feature for a patient suffering from scrub typhus and should be screened for when they do not respond to standard treatment. Prompt treatment with doxycycline can speed up the recovery and shorten the hospital stay.

## Abbreviations

ARDS: Acute respiratory distress syndrome
AKI: Acute kidney injury
SBP: Systolic blood pressure
DBP: Diastolic blood pressure.
